# Interkit Reproducibility of the Indirect Immunofluorescence Assay on HEp-2 Cells Depends on the Immunofluorescence Reactivity Intensity and Pattern

**DOI:** 10.3389/fimmu.2021.798322

**Published:** 2022-01-19

**Authors:** Mônica Jesus Silva, Alessandra Dellavance, Danielle Cristiane Baldo, Silvia Helena Rodrigues, Marcelle Grecco, Monica Simon Prado, Renan Agustinelli, Luís Eduardo Coelho Andrade

**Affiliations:** ^1^ Rheumatology Division, Escola Paulista de Medicina, Universidade Federal de São Paulo, São Paulo, Brazil; ^2^ Research and Development Division, Fleury Medicine and Health Laboratories, São Paulo, Brazil

**Keywords:** autoantibody, antinuclear antibodies, immunofluorescence, HEp-2 cells, autoimmune diseases

## Abstract

**Introduction:**

The indirect immunofluorescence assay on HEp-2 cells (HEp-2/IFA) is used worldwide for screening for autoantibodies to cellular antigens. Cell culture and fixation methods influence the cell distribution of autoantigens and the preservation of epitopes. Therefore, discrepancy of results obtained using different HEp-2/IFA kits (interkit nonreproducibility) is a common phenomenon in the clinical laboratory routine.

**Objective:**

This study evaluated the interkit nonreproducibility of HEp-2/IFA results using samples from patients with systemic autoimmune disease (SAD), nonautoimmune diseases (NAD), and healthy blood donors (HBD).

**Methods:**

Serum from 275 SAD patients, 293 NAD patients, and 300 HBD were processed at 1:80 dilution using four HEp-2 kits according to the manufacturers’ instructions. Interkit reproducibility was determined for positive/negative results and patterns. The agreement of positive/negative results among kits for each sample was determined as the reactivity agreement score (RAS). The pattern reproducibility score (PRS) in each sample was calculated as a function of the number of kits showing equivalent patterns. Qualitative variables and ordinal variables were analyzed by the Chi-square and Mann-Whitney *U* tests, respectively.

**Results:**

A total of 402 samples were nonreactive in all kits and were considered devoid of autoantibodies. Further analysis included the 466 reactive samples (238 SAD, 119 NAD, 109 HBD). Reactivity to the nucleus had the highest interkit reproducibility (RAS = 83.6), followed by the metaphase plate (RAS = 78.9), cytoplasm (RAS = 77.4), and nucleolus (RAS = 72.4). Interkit reproducibility was higher in SAD (RAS = 78.0) than in NAD (RAS = 70.6) and HBD (RAS = 71.3) groups. Samples with strong reactivity (++++/4 and +++/4) had higher interkit reproducibility than those with weak reactivity (+/4). In the SAD group, RAS for nuclear reactivity was 87.5% for strongly reactive samples as opposed to 4.4% for weakly reactive samples, and the same was observed for NAD and HBD samples. The most robust patterns were the centromere AC-3 (PRS = 78.4), multiple nuclear dots AC-6 (PRS = 73.6), nuclear coarse speckled AC-5 (PRS = 71.3), nuclear homogeneous AC-1 (PRS = 67.9), and the reticular cytoplasmic AC-21 (PRS = 68.6).

**Conclusion:**

Interkit nonreproducibility in HEp-2/IFA is prevalent and occurs with the highest frequency with weakly reactive samples. International initiatives with the engagement of *in vitro* diagnostic industry are encouraged to promote the harmonization of the properties and performance of HEp-2/IFA commercial kits.

## Introduction

The indirect immunofluorescence assay on HEp-2 cells (HEp-2-IFA) is the most frequently used method for screening for the presence of a vast array of autoantibodies and was considered the gold standard by a task force commissioned by the American College of Rheumatology (1, 2). The titer of the HEp-2-IFA indicates the relative autoantibody concentration and tends to be higher in patients with systemic autoimmune diseases (SAD) than in nonautoimmune NAD patients and normal individuals with a positive HEp-2-IFA test (3, 4). The immunofluorescence (IF) pattern of the HEp-2-IFA test provides hints for the autoantibody specificities present in the sample (5–10), as it reflects the characteristic topographic distribution of the target antigens along the successive stages of the cell cycle. The HEp-2-IFA patterns hold added clinical value because they indicate autoantibody specificities with clinical relevance (8, 11–15). The homogeneous nuclear pattern (AC-1), for example, suggests the presence of autoantibodies against double-stranded DNA and antinucleosome, which are specific biomarkers of systemic lupus erythematosus (SLE) (16, 17). The centromere nuclear pattern (AC-3) is associated with autoantibodies to the centromere proteins CENP-A, CENP-B, and CENP-C, which are biomarkers of systemic sclerosis and primary biliary cholangitis (18). In contrast, the dense fine speckled nuclear pattern (AC-2) is most frequently observed in healthy individuals and NAD patients but rarely in SAD patients (3, 4, 10, 19). Considering the substantial fraction of the general population with a positive HEp-2-IFA test (20–27), the judicious interpretation of HEp-2-IFA patterns can contribute in the clinical evaluation of a positive test. The recognition of the importance of pattern definition in the HEp-2-IFA test triggered the establishment of standardization recommendations by national expert groups (5–8). In 2014, an international group of specialists launched the International Consensus on ANA Patterns initiative (ICAP), dedicated to standardizing the nomenclature and the clinical relevance of HEp-2-IFA patterns (9, 10). The ICAP website www.anapatterns.org displays the classification algorithm including 30 patterns with their respective alphanumeric AC (anticellular) codes, correspondent images, possible target antigens, and clinical relevance (9, 10).

**Figure 1:**
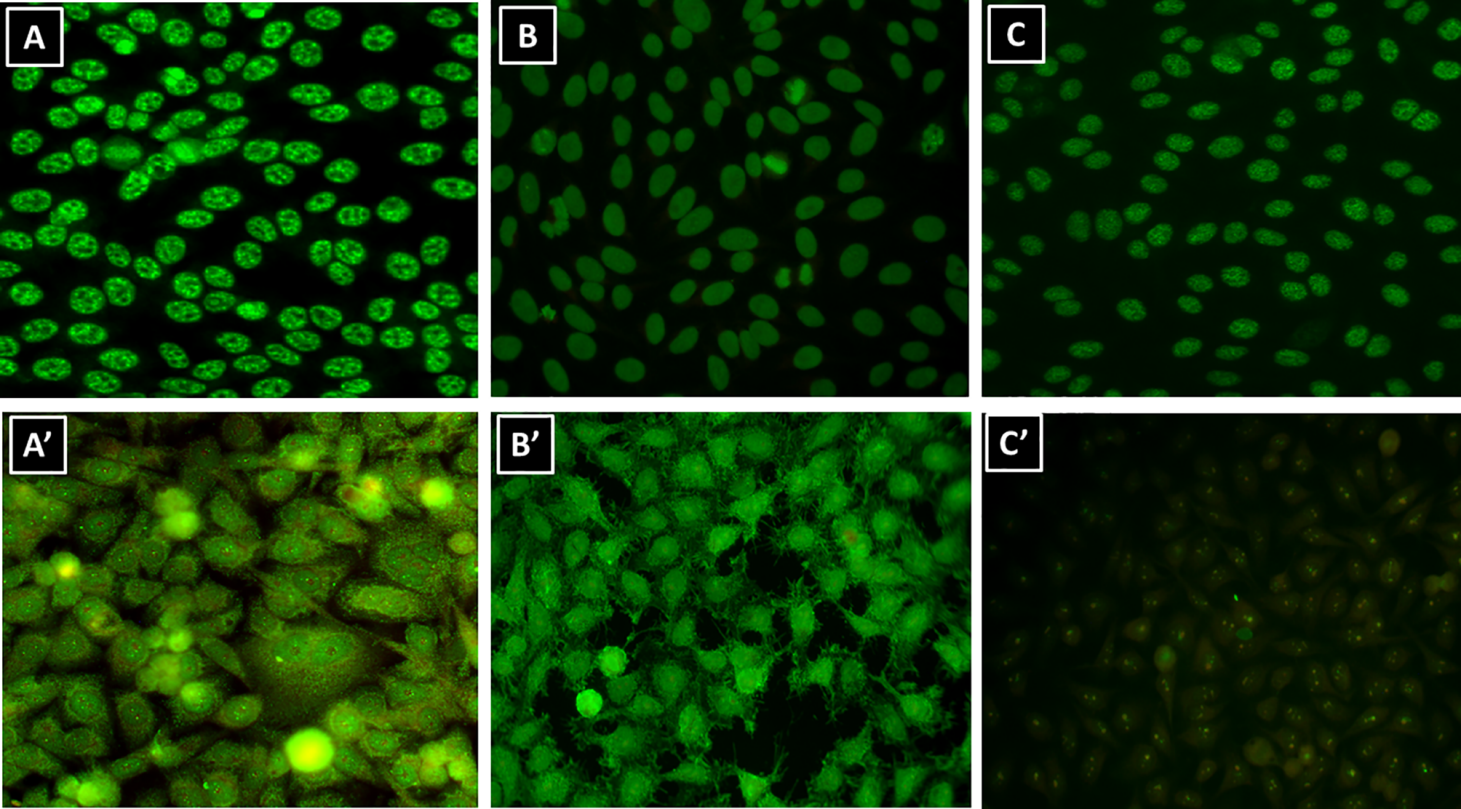
Interkit nonreproducibility of the HEp-2-IFA test. Representative serum samples from the laboratory routine operation diluted 1/160 and processed in different HEp-2-IFA kits according to the manufacturer’s instructions. **(A**, **A’)** Serum #1; **(B**, **B’)** serum #2; **(C**, **C’)** serum #3.

The HEp-2-IFA method has limitations and disadvantages, including subjectivity and dependence on expert analysis of images. One underestimated problem of the HEp-2-IFA method is that some samples produce different results, including different titer and IF patterns, in different kit brands. The interkit nonreproducibility of HEp-2-IFA results is a common phenomenon in the routine of clinical laboratories (**Figure 1**). This scenario may affect the clinical care of patients under investigation of autoimmune diseases. Moreover, the lack of standardization of the methods for culture, permeabilization, and fixation of HEp-2 cells in commercial slides contributes to decreasing the reproducibility of results using different kits and threatens the efforts for harmonization of results between different laboratories. The interkit nonreproducibility phenomenon of the HEp-2-IFA test has been studied previously. Copple et al. compared five HEp-2-IFA kits (28), using samples from 160 patients with assorted SAD, 100 samples from the laboratory routine operation, 100 healthy blood donors (HBD) samples, and 12 reference samples from the Autoantibody Standardization Committee (29). They demonstrated that the interkit nonreproducibility phenomenon varied according to the clinical nature of the samples, with higher reproducibility for samples from HBD and rheumatoid arthritis patients, and lower for scleroderma samples. In addition, they showed that some samples displayed striking divergence in titer. For example, one sample had titers 1/80, 1/320, 1/640, 1/1280, and 1/2560 with the five kits, respectively; four samples were negative with one kit and yielded titers from 1/80 to 1/320 with the other brands (28).

Dellavance et al. compared eight HEp-2-IFA kits using 17 samples with well-defined IF patterns, including nuclear patterns (homogeneous/AC-1, dense fine speckled/AC-2, centromere/AC-3, coarse speckled/AC-5, multiple nuclear dots/AC-6, PCNA-like/AC-13, CENP-F-like/AC-14, nuclear matrix-like coarse speckled, *quasi*-homogeneous, and fine speckled with rare nuclear dots-AC-4/AC-7), nucleolar patterns (homogeneous/C-8, clumpy/AC-9, and punctate/AC-10), cytoplasmic patterns (fine speckled/AC-20 and dense fine speckled/AC-19), and mitotic apparatus patterns (NuMA-like/AC-26 and mitotic fuse/AC-25 (30) The samples were processed and analyzed blindly in three independent expert laboratories. The results show that some patterns (AC-1, AC-2, AC-3, AC-7, AC-8, AC-9, AC-10, AC-19, and nuclear *quasi*-homogeneous) were rather robust in that they were appropriately identified with all kits and in at least two of the three participating laboratories. Some patterns (AC-5, AC-4/AC-7, AC-25, and AC-26) were identified appropriately using all but one kit. Finally, three patterns (AC-13, AC-14, and AC-20) were rather vulnerable as they could be identified appropriately in a minority of the kits in the three laboratories (30). Relevant heterogeneity in results has been also documented when comparing results obtained with different HEp-2 cell kits read in the microscope by expert analysts and also when comparing results obtained by human reading and computer-aided automated readers (31).

The present study provides an in-depth and objective analysis of the phenomenon of interkit nonreproducibility of HEp-2-IFA by establishing semiquantitative reproducibility scores and addressing how this phenomenon varies according to the clinical nature of the sample, the cell compartment stained, the type of HEp-2-IFA pattern, and the intensity of IF reactivity.

## Materials and Methods

### Clinical Samples and HEp-2-IFA Processing

Serum samples from 868 sequential individuals were obtained, including 275 patients with systemic autoimmune disease (SAD), 293 patients with nonautoimmune diseases (NAD), and 300 samples from healthy blood donors (HBD). All subjects provided informed consent and the study was approved by the institutional Ethics Committee at Universidade Federal de Sao Paulo (Protocol #945.320). The SAD group comprised patients with systemic lupus erythematosus (SLE; *n* = 161), systemic sclerosis (SSc; *n* = 28), primary Sjögren syndrome (SjS; *n* = 13), primary biliary cholangitis (PBC; *n* = 30), and autoimmune hepatitis (*N* = 43). All patients met the respective classification or diagnostic criteria (32–36). The NAD group was formed by patients with systemic arterial hypertension (*n* = 74), psychiatric diseases, mainly schizophrenia and bipolar disease (*n* = 75), various cancer malignancies (*n* = 70), and hepatitis C (*n* = 74).

Samples were processed at 1:80 dilution using four HEp-2-IFA kits according to the instructions of the respective manufacturers: Aesku Diagnostics (Oakland, USA), Bion (MBL Bion, Des Plaines, USA), Hemagen (Hemagen Diagnostics, Inc., Columbia, USA), and Inova (Inova Diagnostics, Inc., San Diego, USA). All kits were approved by our quality control assessment, in which a collection of known negative and positive samples with known IFA patterns yielded the expected results. The tests were interpreted by three experienced independent blinded observers under ×400 magnification using an Olympus BX-50 immunofluorescence microscope. Any discrepancy in the reading of the analysts was settled by a group review of the slides, and agreement of at least two of the three observers was obtained for all samples.

### Selection of Reactive Samples and Definition of Scores for Assessing Agreement in Reactivity Using Different HEp-2 Slide Kits

Samples showing no reactivity using the four HEp-2-IFA kits (*n* = 402) were considered devoid of relevant autoantibodies. Conversely, the 466 serum samples showing reactivity to any cell compartment in at least one kit were classified as reactive. Only reactive samples were used throughout the study, and these included 238 SAD samples, 119 NAD samples, and 109 HBD samples.

We analyzed the reproducibility in results obtained with the four kits, for each sample, regarding reactivity separately for each cell compartment. Total agreement was defined as a dichotomous variable that could be classified as positive (positive reactivity using the four HEp-2-IFA kits) or negative (at least one kit differed from the others). In addition, we semiquantified the reproducibility, by developing a reactivity agreement score (RAS) based on the possibilities of agreement among the four kits analyzed: (1) all kits presented similar reactivity (4 × 4); (2) three kits presented similar reactivity and one presented a discordant result (3 × 1); and (3) two kits presented similar reactivity and two were discordant (2 × 2). These three possibilities of agreement received the arbitrary proportional weights of 100, 75, and 50, respectively. The RAS for specific groups of samples was obtained by calculating the mean RAS for all samples in the group of interest. By mathematical definition, the RAS score in any clinical group of samples varies from 50 to 100, and we arbitrarily defined four categories: poor agreement (between 50 and 62.5), moderate agreement (between 62.6 and 75), satisfactory agreement (between 75.1 and 87.5), and excellent agreement (between 87.6 and 100).

The HEp-2-IFA patterns were expressed according to the ICAP nomenclature. We evaluated the robustness of HEp-2-IFA patterns across different HEp-2 kits by assessing the reproducibility of each pattern. A Pattern Reproducibility Score (PRS) was defined as the frequency with which a given pattern is reproducible using the four tested kits in each sample. We assigned arbitrary scores for each of the four possible combinations of results obtained for each sample: (1) the pattern of interest was obtained using the four kits (4 × 4; PRS = 100); (2) the pattern of interest was obtained using three kits (3 × 1; PRS = 67); (3) the pattern of interest was obtained using two kits (2 × 2; PRS = 33); and (4) the pattern of interest was observed using only one kit (1 × 3; PRS = 1). The weighted PRS for each pattern was calculated by obtaining the mean PRS in all samples that presented that pattern in at least one kit. We arbitrarily defined four classes of robustness for the patterns: poor (1≥PRS ≤ 25), moderate (25>PRS ≤ 50), satisfactory (50>PRS ≤ 75), and excellent (75>PRS ≤ 100).

### Characterization of the Interkit Reproducibility of the Intensity of IF-Reactivity Per Cell Compartment in the Three Clinical Groups

The nominal intensity of IF reactivity of each sample was assigned according to the strongest reactivity obtained in any of the kits in a semiquantitative scale as follows: weak (+/4), moderate (++/4), strong (+++/4), and very strong (++++/4). For the analysis of agreement in the intensity of reactivity among the four kits, intensities +/4 and ++/4 were clustered as weak reactivity, while samples with intensity +++/4 and ++++/4 were clustered as strong reactivity. The interkit reproducibility of the intensity in IF reactivity observed for each sample was rated against the nominal intensity of IF reactivity and was assigned as concordant when all kits produced equivalent intensity of reactivity, and discordant when at least one kit produced intensity of reactivity different from the nominal.

### Statistical Analysis

The dichotomous variables were analyzed by the Chi-square test, and ordinal variables were analyzed by the Kruskal-Wallis test and Mann-Whitney *U* test. All data were analyzed using SPSS20.0 software at a significance level of *p* < 0.05.

## Results

As shown in **Table 1**, the 466 reactive samples showed considerable difference in the frequency of positive results according to the four kits, with kit Z yielding the highest frequency and kit Y the lowest frequency of positive results. Among the three clinical groups, there was a higher frequency of positive results in each kit in samples from the SAD group (**Table 1**), with no statistically significant difference in the frequency of reactivity among the HEp-2-IFA kits (89.9% to 94.5%). In contrast, there was significant heterogeneity in the frequency of positive results among the four kits for the NAD group (47.1% to 78.2%) and HBD group (35.8% to 93.6%). This result suggests greater consistency in reactivity across HEp-2-IFA kits in the SAD group as compared to the other groups. **Table 1** also shows that kit Y had the lowest and kit Z had the highest proportions of positive results in all clinical groups: SAD group (89.9% vs. 94.5% positive results, respectively), NAD group (47.1% vs. 78.2%), and HBD group (35.8% vs. 93.6%). It should be noted that the high frequency of positive results in the NAD and HBD clinical groups is expected, as this analysis includes only samples that yielded a positive result in at least one HEp-2 kit.

**Table 1:** Distribution of samples in each clinical group according to the global reactivity in each HEp-2 kit.

Clinical group	Global reactivity^a^	HEp-2 cell kit				*p*-value
		X	Y	Z	W	
**ALL**	**REA**	358 (76.8%)	309 (66.3%)	411 (88.2%)	365 (78.3%)	<0.001
	**NR**	108 (23.2%)	157 (33.7%)	55 (11.8%)	101 (21.7%)	
**SAD**	**REA**	221 (92.9%)	214 (89.9%)	225 (94.5%)	216 (90.8%)	0.108
	**NR**	17 (7.1%)	24 (10.1%)	13 (5.5%)	22 (9.2%)	
	**Total**	238	238	238	238	
**NAD**	**REA**	63 (52.9%)	56 (47.1%)	93 (78.2%)	76 (63.9%)	<0.001
	**NR**	56 (47.1%)	63 (52.9%)	26 (21.8%)	43 (36.1%)	
	**Total**	119	119	119	119	
**HBD**	**REA**	74 (67.9%)	39 (35.8%)	102 (93.6%)	73 (67.0%)	<0.001
	**NR**	35 (32.1%)	70 (64.2%)	7 (6.4%)	36 (33.0%)	
	**Total**	109	109	109	109	

a Global reactivity refers to reactivity in any cell compartment. REA, reactive; NR, nonreactive; SAD, systemic autoimmune disease; NAD, nonautoimmune disease; HBD, healthy blood donors; p-value, inference level of Cochran’s Q test.

### HEp-2-IFA Interkit Reproducibility According to the Clinical Nature of the Samples

Next, we analyzed the interkit reproducibility in global reactivity and reactivity to each cell compartment using samples from each clinical group separately. Due to the low number of samples showing reactivity in the mitotic apparatus, this compartment was not included in this and subsequent statistical analyses of reactivity. As can be seen in **Table 2**, the SAD group presented higher RAS than the other groups, especially regarding the nuclear compartment, which presented RAS of 90 (classified as excellent reproducibility), while the other groups had a satisfactory reproducibility (RAS of 76.7 and 76.0, respectively). Group SAD also achieved higher RAS referent to the cytoplasmic compartment (RAS = 81.8) than the NAD and HBD groups (RAS = 68.9 and RAS = 71.9, respectively). In contrast, the three groups showed similar RAS regarding reactivity to the nucleolus and the metaphase plate. It is noteworthy that groups NAD and HBD showed equivalent agreement in reactivity to all cell compartments.

**Table 2:** Reactivity agreement score (RAS) in each cell compartment according to the clinical group.

Cell compartment	RAS			Comparison between clinical groups		
				*p*-value		
	SAD	NAD	HBD	SAD × NAD	SAD × HBD	NAD × HDB
**Nucleus**	90.0	76.7	76.0	<0.001	<0.001	1.000
**Nucleolus**	72.3	75.0	68.0	1.000	1.000	1.000
**Metaphase plate**	79.5	75.8	79.5	0.598	1.000	1.000
**Cytoplasm**	81.8	68.9	71.9	0.001	0.288	1.000

RAS, reactivity agreement score. Level of statistical inference calculated by the Kruskal-Wallis test.

It is recognized that HEp-2-IFA reactivity tends to occur at higher titer in autoimmune patients than in nonautoimmune patients and normal individuals who have a positive HEp-2-IFA test. This is confirmed in the present cohort, where the SAD group has a low proportion of weak-reactive samples and a high proportion of strong-reactive samples. The opposite was seen in the HBD and NAD groups (**Table 3**). Therefore, we investigated if the highest agreement rates observed in the SAD group could be caused by the higher reactivity intensity in this group, by analyzing the total reactivity agreement rate as a function of the intensity of HEp-2-IFA reactivity in each clinical group. As shown in **Table 3**, the differences among clinical groups and cell compartments, observed in **Table 2**, disappear when comparing samples with equivalent intensity of reactivity. In the SAD group, for example, the total concordance rate in the nuclear compartment was 86.6% for strong-reactivity samples (++++/4) and below 4.5% for weak-reactivity samples (+/4). A similar trend was observed in the NAD and HBD groups for the nuclear compartment and the cytoplasm and metaphase plate compartments for all clinical groups. The nucleolar compartment showed low agreement rates independently of the intensity of reactivity. In general, the samples with strong reactivity in the three clinical groups showed a high agreement rate among different slides, whereas those with low reactivity presented a low agreement rate.

**Table 3:** Distribution of reactive samples according to the reactivity intensity and total reactivity agreement in each cell compartment in the three clinical groups.

Cell compartment and reactivity intensity	Clinical group					
	SAD (*n* = 238)		NAD (*n* = 119)		HBD (*n* = 109)	
	Total agreement^a^	Total	Total agreement	Total	Total agreement	Total
**Nucleus**						
Intensity 1+	1 (4.3%)^b^	23^c^	1 (1.5%)	68	1 (1.0%)	104
Intensity 2+	5 (17.9%)	28	8 (13.8%)	58	8 (12.7%)	63
Intensity 3+	16 (47.1%)	34	9 (45.0%)	20	9 (39.1%)	23
Intensity 4+	129 (86.6%)	149	7 (100.0%)	7	9 (75.0%)	12
Total	151 (64.5%)	234	25 (16.3%)	153	27 (13.4%)	202
**Nucleolus**						
Intensity 1+	0	7	0	15	0	9
Intensity 2+	0	8	1(16.7%)	6	1 (33.3%)	3
Intensity 3+	1 (10.0%)	10	2 (66.7%)	3	0	2
Intensity 4+	1 (25.0%)	4	0	0	0	0
Total	2 (6.9%)	29	3 (12.5%)	24	1 (7.1%)	14
**Plate**						
Intensity 1+	0	5	0	15	0	7
Intensity 2+	0	13	1 (7.7%)	13	0	8
Intensity 3+	1 (3.7%)	27	2 (15.4%)	13	4 (40.0)	10
Intensity 4+	50 (54.9%)	91	3 (60.0%)	5	4 (57.1)	7
Total	51 (37.5%)	136	6 (13.0%)	46	8 (25.0)	32
**Cytoplasm**						
Intensity 1+	0	15	0	21	0	5
Intensity 2+	1 (5.6%)	18	0	18	0	8
Intensity 3+	7 (21.2%)	33	1 (8.3%)	12	0	2
Intensity 4+	23 (62.2%)	37	1 (33.3%)	3	0	0
Total	31 (33.3%)	93	2 (3.7%)	54	0	15
**Mitotic apparatus**						
Intensity 1+	0	1	0	2	0	4
Intensity 2+	0	0	0	6	0	1
Intensity 3+	0	0	0	1	0	0
Intensity 4+	2 (66.7%)	3	0	0	0	0
Total	2 (50.0%)	4	0	9	0	5

a Total reactivity agreement implies that reactivity was observed in the four slide brands.

b Number of samples showing total agreement.

c Total number of samples in each category of reactivity intensity.

### Robustness of the Various HEp-2-IFA Patterns Using Different Kits

**Figure 2:**
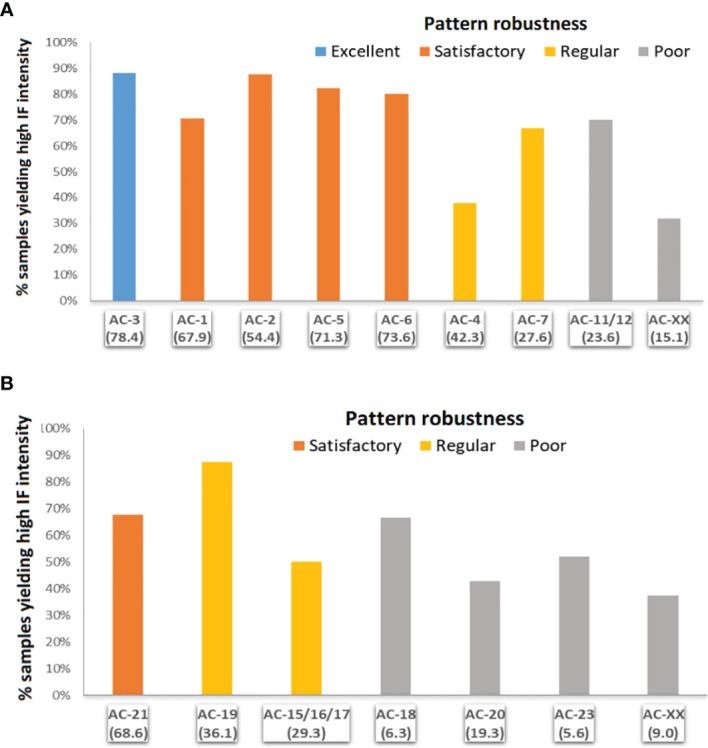
Robustness of the various HEp-2-IFA patterns using different HEp-2 slide brands according to intensity of immunofluorescence intensity. **(A)** Nuclear patterns: AC-1, homogeneous; AC-2, dense fine speckled; AC-3, centromere; AC-4, fine speckled; AC-5, coarse speckled; AC-6, multiple nuclear dots; AC-7, few nuclear dots; AC-11/12, nuclear envelope; AC-XX, atypical. **(B)** Cytoplasmic patterns: 15/16/17 (fibrillary); AC-18, rods and rings; AC-19, dense fine speckled; AC-20, fine speckled; AC-21, mitochondria like. Robustness defined according to the pattern reproducibility score (PRS): excellent (75>PRS), satisfactory (50>PRS ≤ 75), moderate (25>PRS ≤ 50), poor (1≥PRS ≤ 25).

We assessed the robustness of patterns by calculating the PRS, defined according to the frequency with which a given pattern is observed using the four kits in all samples that presented that pattern in at least one kit. In general, nuclear patterns were more robust than cytoplasmic patterns in terms of reproducibility using different HEp-2 kits (**Table 4**). Among the nuclear patterns, the reproducibility was classified as excellent for the AC-3 pattern, satisfactory for AC-6, AC-5, AC-1, and AC-2 patterns, moderate for AC-4 and AC-7 patterns, and poor for AC-11/AC-12 and AC-XX patterns (**Table 4**; **Figure 2A**). In general, the cytoplasmic patterns had lower PRS values, with reproducibility classified as satisfactory for AC-21, moderate for AC-19 and the cytoskeleton (AC-15, AC-16, and AC-17) patterns, and poor for AC-20, AC-18, AC-23, and AC-XX patterns (**Table 4**; **Figure 2B**).

**Table 4:** Robustness of HEp-2-IFA patterns is associated with strong reactivity in the indirect immunofluorescence assay.

Patterns (AC codes)	Pattern robustness score (PRS)^a^	Immunofluorescence reactivity		
		Strong	Weak	Total
		N (%)	N (%)	
**AC-1**	67.9	55 (70.5%)	23 (29.5%)	78
**AC-2**	54.4	14 (87.5%)	2 (12.5%)	16
**AC-3**	78.4	15 (88.2%)	2 (11.8%)	17
**AC-4**	42.3	100 (37.9%)	164 (62.1%)	264
**AC-5**	71.3	37 (82.2%)	8 (17.8%)	45
**AC-6**	73.6	4 (80.0%)	1 (20.0%)	5
**AC-7**	27.6	14 (66.7%)	7 (33.3%)	21
**AC-11/12**	23.6	7 (70.0%)	3 (30.0%)	10
**AC-XX (nucleus)**	15.1	15 (31.9%)	32 (68.1%)	47
**AC-15/16/17**	29.3	4 (50%)	4 (50%)0	8
**AC-18**	6.3	4 (66.7%)	2 (33.3%)	6
**AC-19**	36.1	12 (85.7%)	2 (14.3%)	14
**AC-20**	19.3	3 (42.9%)	4 (57.1%)	7
**AC-21**	68.6	40 (67.8%)	19 (32.2%)	59
**AC-23**	5.6	4 (57.1%)	3 (42.9%)	7
**AC-XX (cytoplasm)**	9.0	3 (37.5%)	5 (62.5%)	8

Immunofluorescence reactivity: strong (+++/4 and ++++/4); weak (+/4 and ++/4).

a The reproducibility of each pattern using different HEp-2 kits is displayed as the PRS (see Material and Methods) and expresses the robustness of the respective HEp-2-IFA pattern. Robustness of HEp-2-IFA patterns was arbitrarily classified as excellent (75>PRS), satisfactory (50>PRS ≤ 75), moderate (25>PRS ≤ 50), and poor (1≥PRS ≤ 25).

We then investigated if the robustness of HEp-2-IFA patterns was associated with the intensity of IF reactivity. In general, patterns with higher PRS tended to present a higher frequency of samples with strong IF reactivity (**Table 4**). Thus, among the nuclear patterns, those with excellent and satisfactory robustness (AC-3, AC-1, AC-5, and AC-6) had the highest frequency of samples with strong IF reactivity. In contrast, patterns with moderate and poor robustness (AC-4, AC-7, AC-11/12, AC-XX) presented a lower frequency of samples with strong IF reactivity (**Figure 2A**). However, there were some exceptions to this trend, e.g., the AC-2 pattern showed the lowest PRS (54.2) among the patterns with satisfactory robustness (the others varied from 67.9 to 78.4) but showed the highest frequency of samples with strong IF reactivity in this group. Similarly, the cytoplasmic pattern AC-21, classified as satisfactory robustness (PRS = 68.6), had a lower frequency of samples with strong IF reactivity than the pattern AC-19, classified as moderate robustness (PRS = 36.1) (**Figure 2B**). This dual behavior indicates that the intensity of IF reactivity tends to favor reproducibility, but some patterns have intrinsic characteristics of robustness independent of the intensity of IF reactivity.

## Discussion

The present study investigated how the interkit nonreproducibility phenomenon of the HEp-2-IFA test varies according to the clinical nature of the sample, the cell compartment stained, the type of HEp-2-IFA pattern, and the intensity of IF reactivity. Thus, we established semiquantitative scores for determining the interkit nonreproducibility phenomenon in samples from different clinical groups, with reactivity to different cell compartments, different IF patterns, and different IF-reactivity intensity. The interkit nonreproducibility phenomenon was investigated systematically by analyzing 466 HEp-2-IFA-reactive samples from SAD patients, NAD patients, and HBD. The interkit reproducibility was determined according to two perspectives. The total agreement score is a very stringent binary parameter in which one discordant result using one of the kits would assign a nonreproducibility status. Therefore, we also assessed the interkit reproducibility in a more judicious and balanced way by establishing the RAS and PRS scores, which allow the determination of increasing intermediate degrees of reproducibility. From this perspective, we could semiquantify the interkit reproducibility phenomenon according to the clinical nature of the sample, the reactivity to each cell compartment, the HEp-2-IFA pattern, and the intensity of IF reactivity.

We demonstrated that reproducibility was greater with samples from SAD patients and samples reactive with the nucleus, and this was associated with the strongest IF reactivity in these groups of samples. In other words, the SAD group and the nuclear compartment showed higher reproducibility precisely because they have a higher frequency of samples with strong IF reactivity. Some patterns had higher reproducibility than others did, and this was again partially associated with the intensity of IF reactivity of the samples. AC-3, for example, was the most robust pattern (highest PRS) and presented the highest frequency of samples with strong IF reactivity. However, for some IF patterns, the reproducibility was not fully dependent on the intensity of IF reactivity. The nuclear AC-2 pattern, for example, had lower reproducibility but a higher frequency of samples with strong IF reactivity than the AC-1 pattern. In other words, the AC-1 pattern was more robust than the AC-2 pattern, independently of the intensity of IF reactivity. These observations indicate that weak IF reactivity of the samples contributes to poor interkit reproducibility of results, but intrinsic characteristics of some patterns affect their reproducibility in different kits independently of the IF-reactivity intensity.

We observed considerable differences in the frequency of positive results obtained with the four kits in the three clinical groups, with kit Z systematically showing the highest frequency and kit Y showing the lowest frequency of positive results. It should be noted that the difference between kits Y and Z was less noticeable in the SAD group than in the NAD and HBD groups. As the interkit nonreproducibility phenomenon was especially evident in samples with weak IF reactivity, it is possible that the lot of kit Z used in this study yielded inappropriately high sensitivity. In this report, the HEp-2 kits were coded and the brand names were not disclosed in the results because we understand that there may be lot-to-lot variation in any immunoassay and therefore the characteristics observed in this study cannot be unconditionally attributed to each kit brand. However, we issued a report to each manufacturer disclosing the identity of their respective kits.

In general, samples from SAD patients showed higher reproducibility rates for global reactivity and cell compartment reactivity, especially in the nuclear, cytoplasmic, and metaphase plate compartments. Intriguingly, the reactivity with the nucleolus showed lower rates of reproducibility than the other cellular compartments did in the three clinical groups. As mentioned above, for all clinical groups, the reproducibility was higher in samples with strong IF reactivity, and this analysis shows that the higher number of samples with strong IF reactivity accounted for the higher reproducibility rates obtained with samples from the SAD group. The same applies to the interkit reproducibility of reactivity with the nucleus, cytoplasm, and metaphase plate. However, the interkit reproducibility of reactivity with the nucleolus was poor even in samples with strong IF reactivity and in all clinical groups. This observation suggests that nucleolar autoantigens are particularly susceptible to peculiarities in the methods for culture, permeabilization, and fixation of HEp-2 cells used by the different manufacturers.

The monolayer of HEp-2 cells on the glass slides allows the detection of dozens of autoantibodies against different autoantigens, and the IF patterns reflect the topographic distribution of these autoantigens as well as their behavior throughout the cell cycle. Therefore, the HEp-2-IFA patterns provide a preliminary indication of the possible autoantibodies present in the test sample (3–14, 37). The recognition of this important aspect of HEp-2-IFA patterns has stimulated a progressive international commitment to harmonize the nomenclature of HEp-2-IFA patterns, culminating with ICAP international initiative (9, 10). However, cell culture conditions and fixation methods influence the cell distribution of autoantigens and the preservation of epitopes of interest (37–40). There are dozens of HEp-2-IFA kits available in different parts of the world and each manufacturer uses a particular methodology for growing, permeabilizing, and fixing the cells onto the slides. In addition, there is heterogeneity in the proprietary buffers and conjugates from each manufacturer. The heterogeneity and lack of standardization in the preparation of kits by manufacturers contribute to the discrepancy of results obtained using different HEp-2-IFA kits. Previous studies provide an experimental technical basis to explain the inconsistency of results between different HEp-2 kits, pointing out that cell fixation and permeabilization protocols are capable of modifying the structure and composition of cell compartments, the size of nuclei and nucleoli, and the availability of epitopes for recognition by autoantibodies (40–44).

The present study confirms previous findings on the phenomenon of interkit nonreproducibility of HEp-2-IFA results (28, 30, 45, 46) and shows that this phenomenon is especially frequent in samples from normal individuals and patients with nonautoimmune diseases. In addition, we demonstrated that this phenomenon affects particularly samples with low IF intensity as well as some specific patterns. This is relevant for the routine HEp-2-IFA testing in that the majority of samples from nonautoimmune patients derived from a low positive predictive value scenario have low-to-moderate titer. Thus, samples with low IF intensity might be considered for confirmation in at least one additional HEp-2-IFA kit.

It is appropriate to recognize that discrepancy in results obtained with different kits is a common observation also for other types of immunoassays, such as ELISA and chemiluminescence. The literature contains several studies demonstrating discrepancy in the results of serum samples submitted to comparison in different commercial immunoassays using the same methodological platform (47–50). Solid-phase immunoassays (SPIA) are widely applied in the determination of autoantibodies of clinical relevance and there are multiple brands of SPIA kits approved by regulatory agencies. However, there are disturbingly high rates of disagreement in results obtained with different kits (47, 49, 50). Costa-Pereira et al. tested serum samples from 144 patients with autoimmune rheumatic diseases and 121 individuals with nonautoimmune diseases using traditional double immunodiffusion and seven SPIA kits for rheumatic disease-related autoantibodies (U1-RNP, SS-A/Ro, SS-B/La, Sm, Jo-1, and Scl-70) (51). Regarding the clinical diagnosis, SPIA kits were more sensitive and double immunodiffusion was more specific for all autoantibodies. Remarkably, there was a high rate of disagreement among the different SPIA kits regarding positive results for all the autoantibodies tested. For example, the sensitivity for anti-SS-A/Ro in patients with rheumatic diseases varied from 21% to 78% in the different kits (51). Similar disagreements among different kits for rheumatic disease-related autoantibodies were reported by Jaskowski et al. and Van Duijnhoven et al. (49, 50). Provided that each manufacturer uses a peculiar array of reagents for the preparation of kits and adjusts the cutoff for positive results with a particular collection of serum samples, it is no surprise that there is a high rate of disagreement among kits (47–51). The problem of interkit nonreproducibility is a generalized phenomenon in immunoassay testing that also affects the HEp-2-IFA method, particularly concerning the IFA pattern definition.

One limitation of this study is that we used only four HEp-2-IFA kit brands, and this was conditioned by the difficulty in processing and analyzing circa 900 samples in many kits, as well as the consequent budget constraints. However, we used kits that are among the most frequently used, according to the External Quality Assessment program of the College of American Pathologists. We believe that the inclusion of additional kit brands would increase the possibility of identifying nonreproducibility of results, but this would not affect the general findings and conclusions of the study. The results obtained with the four kits already demonstrate clearly that interkit nonreproducibility in HEp-2-IFA is a prevalent phenomenon. This study did not address the nonreproducibility among lots of the same kit brand and this point should be addressed in future studies. We did not determine the titer of the samples; instead, the IF-reactivity intensity was determined in a subjective 4-point semiquantitative assessment. However, this semiquantitative assessment was sufficient to demonstrate consistently that interkit nonreproducibility was more prominent in samples with weak IF reactivity.

To the best of our knowledge, there is no official technical recommendation for the culture, permeabilization, and fixation of HEp-2 cells used in HEp-2-IFA kits. Each manufacturer uses proprietary protocols contributing substantially to the heterogeneity in the performance of the various HEp-2-IFA kits. As documented in the present study, one can easily imagine how the interkit nonreproducibility phenomenon can have a considerable clinical impact and generate divergence in the interpretation of results from different laboratories, influencing the sensitivity, specificity, and positive/negative predictive values of the HEp-2-IFA test. Considering that part of this phenomenon results from the intrinsic heterogeneity of HEp-2-IFA kits, we suggest that international autoantibody standardization initiatives establish a task force, with the involvement of *in vitro* diagnostic company scientists, aiming to elaborate official guidelines for harmonization in the manufacturing of HEp-2-IFA kits.

## Data Availability Statement

The original contributions presented in the study are included in the article/supplementary material. Further inquiries can be directed to the corresponding author.

## Ethics Statement

The studies involving human participants were reviewed and approved by the UNIFESP Research Ethics Committee (CEP-UNIFESP). Written informed consent to participate in this study was provided by the participants’ legal guardian/next of kin.

## Author Contributions

MS, AD, and LA designed the study. MP and RA selected the clinical samples. SR, AD, MG, and LA proceeded to the HEp-2-IFA analysis. The manuscript was drafted by MS and edited by LA. All authors contributed to the article and approved the submitted version.

## Funding

This study was partially supported by Fleury Medicine and Health Laboratories, São Paulo, Brazil. LECA receives a research grant (PQ-1D 310334/2019-5) from the Brazilian National funding agency CNPq.

## Conflict of Interest

The authors declare that the research was conducted in the absence of any commercial or financial relationships that could be construed as a potential conflict of interest.

## Publisher’s Note

All claims expressed in this article are solely those of the authors and do not necessarily represent those of their affiliated organizations, or those of the publisher, the editors and the reviewers. Any product that may be evaluated in this article, or claim that may be made by its manufacturer, is not guaranteed or endorsed by the publisher.
